# Supplementary material for: Canadian Physicians’ Use of Intramuscular Botulinum Toxin Injections for Shoulder Spasticity: A National Cross-Sectional Survey
Source: Toxins (Basel). 2023 Jan 10;15(1):58. doi: 10.3390/toxins15010058 (PMC9866374; doi:10.3390/toxins15010058)
Supplement: Supplementary file 1 [file toxins-15-00058-s001.zip › toxins-2085259-supplementary.pdf]

toxins

MDPI

Survey of Canadian physicians' practice patterns in the management of shoulder spasticity and hemiplegic shoulder pain.

PREAMBLE: For the purpose of this survey, early intervention is defined as management within 12 weeks. The key muscles addressed for the shoulder are Pectoralis major, Pectoralis Minor, Subscapularis, Latissimus Dorsi, Teres Major, Deltoid and biceps.
PSS denotes Post Stroke spasticity **CRPS** denotes Complex Regional Pain Syndrome. **HSP** hemiplegic shoulder pain. **DNB** Diagnostic Nerve Block. **BoNT**. Botulinum Neurotoxin.

Q1. Province / territory of practice

Q2. Specialty/Practice

- Physical Medicine & Rehabilitation
- Neurology
- Pediatrics
- Orthopedic Surgery
- Plastic Surgery
- Other

Q3. In what setting do you see patients for spasticity assessment and management? Please select all that apply:

Outpatient clinic

Acute care setting

Rehabilitation unit

Other:

Q4. How many years have you been using BoNT intramuscular injections for spasticity management?

- 0-9
- 10-19
- 20-29
- >30

Q5. Do you treat children or adults with spasticity?

- ☐ Adults only
- ☐ Children only
- ☐ Both children and adults

Q6. What spasticity population(s) do you service? Select all that apply:

- ☐ Stroke
- ☐ Traumatic Brain Injury
- ☐ Cerebral Palsy
- ☐ Multiple Sclerosis
- ☐ Spinal cord injury
- ☐ Other and idiopathic (e.g. hereditary spastic paraparesis, ALS)

The following questions address shoulder girdle spasticity.

Q7. Which positions do you commonly see in the hemiplegic or spastic shoulder?
Jorg Wissel et al described 5 common patterns of upper limb spasticity. All 5 include shoulder internal rotation, and 4/5 include adduction.

Arm spasticity	Pattern I	Pattern II	Pattern III	Pattern IV	Pattern V
	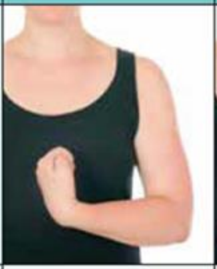	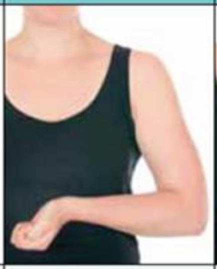	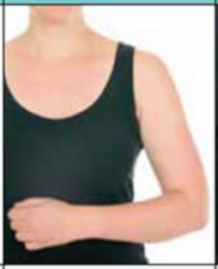	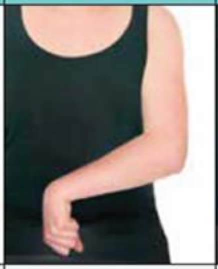	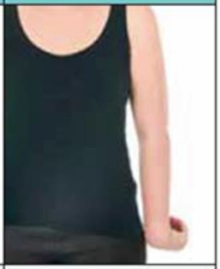
**Shoulder**	*Internal rotation/ adduction*	*Internal rotation/ adduction*	*Internal rotation/ adduction*	*Internal rotation/ adduction*	*Internal rotation/ retroversion*
**Elbow**	*Flexion*	*Flexion*	*Flexion*	*Flexion*	*Extension*
**Forearm**	*Supination*	*Supination*	*Neutral*	*Pronation*	*Pronation*
**Wrist**	*Flexion*	*Extension*	*Neutral*	*Flexion*	*Flexion*

Hefter H, Jost WH, Reissig A, Zakine B, Bakheit AM, Wissel J. Classification of posture in poststroke upper limb spasticity: A potential decision tool for botulinum toxin A treatment? Int J Rehabil Res. 2012;35(3):227–33

Q8. When considering POST STROKE SPASTICITY (PSS), how often do you observe that the shoulder is held in the internally rotated and adducted position?

- 0-25%
- 25-50%
- 50-75%
- 75-100%

Q9. When considering other causes of spasticity, including MS, SCI, and CP,, how often do you observe that the shoulder is held in the internally rotated and adducted position?

- 0–25%
- 25–50%
- 50–75%
- 75–100%

Q10. Do you inject the muscles of the shoulder girdle with BoNT for spasticity management

The muscles that comprise the function of the shoulder girdle are the trapezius muscle (upper, middle, and lower), levator scapulae muscle, rhomboid muscles (major and minor), serratus anterior muscle, and pectoralis muscle group, rotator cuff muscles, latissimus dorsi, teres major, biceps, deltoid.

- Yes
- No

[If yes to the question above]

Q11. Which muscles do you inject with BoNT for adducted internally rotated shoulder spasticity? Please rank in order of frequency. (1 denotes the most frequent). Rank as many as apply to your practice.

- Pectoralis Major
- Pectoralis Minor
- Subscapularis
- Latissimus Dorsi
- Teres Major
- Biceps
- Other

Q12. How often do you inject the following shoulder muscles with BoNT for shoulder adduction and internal rotation? 1) Always 2) Often 3) Sometimes 4) Rarely 5) Never

- Pectoralis Major
- Pectoralis Minor
- Subscapularis
- Latissimus Dorsi
- Teres Major
- Biceps
- Other

Q13. When considering upper extremity PSS, how often do you identify problematic spasticity that requires management with BoNT in the shoulder as part of your plan? (i.e., hygiene issues, difficulty with dressing, affecting ADLs, skin breakdown, pain, mobility and reduced range of motion)

- Often
- Sometimes
- Seldom
- Never

Q14. When considering other causes of upper limb spasticity with adduction and internal rotation, including MS, SCI, and CP, how often do you identify problematic spasticity that requires management with BoNT in the shoulder as part of your plan.

- Often
- Sometimes
- Seldom
- Never

Q15. How often do you include shoulder muscles in your first round of management with BoNT if adduction and/or internal rotation spasticity is identified?

- Often
- Sometimes
- Seldom
- Never

Q16. What dose ranges in units do you inject for each muscle?

Muscle	Botox Onabutulinum toxin A	Dysport Abobotulinum toxin A	Xeomin Incobotulinum toxin A	Other	Other
Pectoralis Major					
Pectoralis Minor					
Subscapularis					
Latissimus Dorsi					
Teres Major					
Deltoid					
Other					

Q17. What is the minimum Modified Ashworth Scale score you will inject BoNT for the shoulder?

- 0- No increase in muscle tone
-
- 1- Slight increase in muscle tone, manifested by a catch and release or by minimal resistance at the end of the range of motion when the affected part(s) is moved in flexion or extension
-
- 1+- Slight increase in muscle tone, manifested by a catch, followed by minimal resistance throughout the remainder (less than half) of the ROM
-
- 2- More marked increase in muscle tone through most of the ROM, but affected part(s) easily moved
-

- 3- Considerable increase in muscle tone, passive movement difficult
-
- 4- Affected part(s) rigid in flexion or extension

Q18. Do you ever inject BoNT for shoulder abduction spasticity?

- No
- Yes

Q 18b. If yes, which muscles do you inject? Select all that apply.
- Deltoid
- Latissimus Dorsi
- Rhomboids
- Biceps
- Supraspinatus
- Other:

Q19. When you inject the shoulder muscles with BoNT for adduction and internal rotation, what are your goals? Select all that apply:

- Reduce spasticity as measured with the Modified Ashworth Scale or similar
- Increase range of motion
- Reduce pain
- Achieve a patient or caregiver goal such as using the Goal Attainment Scale.
- Other:

Q20. What target muscle localization method(s) do you use for BoNT injection around the shoulder girdle? Please select all that apply:

- Anatomical landmarks only
- Electromyography (EMG)
- Electrical stimulation
- Ultrasound
- Other:

Q21. Do you ever grab the wad of muscles for the pectoralis or latissimus dorsi and inject while grasping?

- Yes
- No

Q22. Please list the number of lung punctures that have occured with shoulder muscle injections that caused a pneumothorax for you personally?

0
1
2
3
4
>4

Q23. Do you use Phenol or Alcohol for Shoulder adduction or internal rotation spasticity?

- No
- Yes

Q23b. If yes, which nerves do you target? Select all that apply:

- Lateral Pectoral Nerve
- Medial Pectoral Nerve
- Thoracodorsal nerve
- Subscapular nerve
- Other:

Q24. If you suspect contracture causing shoulder internal rotation and/or adduction, have you referred such patients for surgical release?

- Often
- Sometimes
- Seldom
- Never

Q: What improved outcomes have you observed to the use of BoNT injections on patients with shoulder spasticity?

Open Response:

Q: What complications have you observed to the use of BoNT injections on patients with shoulder spasticity?

Open Response:

Q25. What are barriers to the use of BoNT for patients with shoulder spasticity as compared to other upper extremity regions. Select all that apply:

- Financial – physician/clinic resources
- Financial – patient resources
- Clinic not equipped with necessary equipment
- Clinician time constraints (e.g., clinical practice too busy)
- Risk of adverse events
- Lack of evidence in the literature
- Lack of effectiveness from clinical experience
- Lack of interdisciplinary care
- Patient does not want to have BoNT treatment
- No barriers
- Off-Label
- Other: (text box option)

Q: What improved outcomes have you observed to the use of phenol on patients with shoulder spasticity?

Open Response:

Q: What complications have you observed to the use of phenol on patients with shoulder spasticity?

Open Response:

Q26. If you use phenol, what are any barriers to the use in patients with shoulder spasticity as compared to other upper extremity regions

- Financial – physician/clinic resources
- Financial – patient resources
- Clinic not equipped with necessary equipment
- Clinician time constraints (e.g., clinical practice too busy)
- Risk of adverse events
- Lack of evidence in the literature
- Lack of effectiveness from clinical experience
- Lack of interdisciplinary care
- Patient does not want to have BoNT treatment
- No barriers
- Off-Label
- Other: (text box option)

Q27. The next section deals with early intervention of the hemiplegic shoulder in Post Stroke Spasticity (PSS) only.

Hemiplegic shoulder pain (HSP) is the most common pain disorder after stroke and one of the four most common complications of stroke. It is known that permanent loss of range of motion can occur within three to six weeks. According to the 2019 Canadian Stroke Best Practice Recommendations (CSBPR) there is a lack of evidence supporting individual treatments.

Q28. Do you assess and treat patients for spasticity within twelve weeks of stroke?

○ No – (no need to continue)

○ Yes

Q28b. If yes what is the minimum time (post-stroke) that you would treat these patients for spasticity with BoNT?

- <1 week
- 1 week
- 2 weeks
- 3 weeks
- 4 weeks
- 5 weeks
- 6 weeks
- >6 weeks
-

Q28c. If yes what is the minimum time (post-stroke) that you would treat these patients for spasticity with phenol or alcohol?

- <1 week
- 1 week
- 2 weeks
- 3 weeks
- 4 weeks
- 5 weeks
- 6 weeks
- >6 weeks

Q29. Do you assess and treat hemiplegic shoulder pain (HSP) in the first twelve weeks after stroke?

◯ Yes

◯ No – no need to complete

Q29c. If yes how soon after stroke would you see on average?

- <1 week
- 1 week
- 2 weeks
- 3 weeks
- 4 weeks
- 5 weeks
- 6 weeks
- >6 weeks

Q30. The shoulder hand syndrome after stroke is a well described variant of Complex Regional Pain Syndrome (CRPS) with an estimated incidence of up to 48.8% in the first 28 weeks post-stroke. Do you assess for and treat this condition?

◯ Yes

◯ No

Q31. What is your treatment for post-stroke CRPS? Select all that apply:

◯ Physical therapies

◯ Bracing

◯ Intraarticular steroids

◯ Opioids

◯ Oral corticosteroids NSAIDS

◯ Neuropathic pain medications

◯ Bisphosphonates

◯ BoNT injections

◯ Other medications/interventions:

Q32. How confident are you that you can differentiate the causes of HSP in PSS that presents with a shoulder that is stiff and hard to move, including adhesive capsulopathy, rotator cuff pathology, glenohumeral pathology, myofascial pain, spasticity, contracture, heterotopic ossification and CRPS?

1- Very confident
2- Somewhat confident
3- Not confident.
4- Never thought to differentiate

Q33. Do you perform diagnostic nerve blocks on muscles of the shoulder girdle to differentiate spasticity from contracture or primary shoulder pathology?

- No
- Yes

Q 33b. If yes:

- Lateral Pectoral Nerve
- Medial Pectoral Nerve
- Thoracodorsal nerve

Q34. Do you offer subacromial or glenohumeral injections of corticosteroids and anesthetics for HSP?

- Often
- Sometimes
- Seldom
- Never

Q35. Do you offer suprascapular nerve blocks for HSP?

- Often
- Sometimes
- Seldom
- Never

Q36. Do you offer suprascapular nerve radiofrequency neurotomy or cryoneurotomy for HSP?

- **Often**
- **Sometimes**
- **Seldom**
- **Never**

Q37. LAST QUESTION! We are very interested in your clinical experience, which can make a valuable addition to the literature, especially given the limited evidence for the management of shoulder spasticity and HSP. Please use the text box below to add any tips/pearls of wisdom regarding your experience with management of the shoulder.

____

____

____

____

____

Many thanks for your participation. Please do not hesitate to forward the survey to any colleagues you know.
